# Difficult Cases

**Published:** 1886-10-30

**Authors:** 


### DIFFICULT CASES.

### Care of Epileptics.

M. G. E. writes: "The parents of a lad subject to epileptic fits, followed by uncontrollable outbursts of temper, are most anxious to get him admitted into some institution where he will be well cared for and medically treated, as he is getting beyond their control. Some of your numerous correspondents would confer a favour by affording such information as will secure the object desired."

*** M. G. E. should write to the Medical Superintendent, of the Earlswood Idiot Asylum or the Royal Albert Asylum, Lancaster.

### An Intractable Child.

Miss Sarah Jacombs, of 33, Abbey Road, St. John's Wood, N.W., who is recommended by G. W. Potter, Esq., M.D., writes: "My attention has been drawn to the requirements of F. F. G., as stated in The Hospital of October 2nd, and as I have had some experience in several cases, and I should be able to give individual attention, I should be willing to receive the child if terms could be arranged. I had one case of a little girl 11 or 12 years old, who in infancy had been the subject of epilepsy, and was still suffering from its effects. She was at times unmanageable and wilful, but my sister, who specially undertook the charge of her, exercised such tact and discipline that the child's health and disposition greatly improved, the mother expressed her satisfaction and gratitude, and the child became quite attached to us. If F. F. G. will, therefore, communicate with me, I shall be able to judge whether the case is one we can undertake, as we know by long experience that it is by suitable management alone one can expect success in these cases."

### A Case of Deformity.

The Matron of the Blackheath Cottage Hospital writes: "Can anyone recommend a home (permanent) for a little girl of six years, who has lost her right arm, and is slightly deformed? The mother is a widow in ill-health, and very poor circumstances. The child is bright and intelligent. Payment to a small extent would be guaranteed. The child was a patient here for a long time."

"A Colonel's Wife" writes: "Will you kindly tell me what hospital will be the best for a child of ten with a curvature of the spine? The poor little thing has tried two or three provincial hospitals, and Dr. B. (one of our doctors) is most anxious for her to try London. She has had an abscess in her hip, and is very weak, but he still wishes her to go. I am sorry to trouble you, but I don't know what steps to take about getting her to a Children's Hospital, or one for her complaint. They all come to me for advice, and I am so busy, as we have a great deal of typhoid just now."

